# A Computational Study of the Effects of Syk Activity on B Cell Receptor Signaling Dynamics

**DOI:** 10.3390/pr3010075

**Published:** 2015-02-11

**Authors:** Reginald L. McGee, Mariya O. Krisenko, Robert L. Geahlen, Ann E. Rundell, Gregery T. Buzzard

**Affiliations:** 1 Department of Mathematics, Purdue University, 150 N. University St., West Lafayette, IN 47907, USA; 2 Department of Medicinal Chemistry and Molecular Pharmacology, Purdue University, 201 S. University Street, West Lafayette, IN 47907, USA; 3 Weldon School of Biomedical Engineering, Purdue University, 206 S. Martin Jischke Drive, West Lafayette, IN 47907, USA

**Keywords:** B cell signaling, signal transduction, mutant Syk, computational modeling, cell response

## Abstract

The kinase Syk is intricately involved in early signaling events in B cells and is required for proper response when antigens bind to B cell receptors (BCRs). Experiments using an analog-sensitive version of Syk (Syk-AQL) have better elucidated its role, but have not completely characterized its behavior. We present a computational model for BCR signaling, using dynamical systems, which incorporates both wild-type Syk and Syk-AQL. Following the use of sensitivity analysis to identify significant reaction parameters, we screen for parameter vectors that produced graded responses to BCR stimulation as is observed experimentally. We demonstrate qualitative agreement between the model and dose response data for both mutant and wild-type kinases. Analysis of our model suggests that the level of NF-*κ*B activation, which is reduced in Syk-AQL cells relative to wild-type, is more sensitive to small reductions in kinase activity than Erkp activation, which is essentially unchanged. Since this profile of high Erkp and reduced NF-*κ*B is consistent with anergy, this implies that anergy is particularly sensitive to small changes in catalytic activity. Also, under a range of forward and reverse ligand binding rates, our model of Erkp and NF-*κ*B activation displays a dependence on a power law affinity: the ratio of the forward rate to a non-unit power of the reverse rate. This dependence implies that B cells may respond to certain details of binding and unbinding rates for ligands rather than simple affinity alone.

## 1. Introduction

Signaling through the B cell receptor (BCR) involves an intricate network of molecular reactions necessary for B cells to generate an immune response. The signaling network involves a variety of proteins including kinases and phosphatases and is particularly dependent on the protein-tyrosine kinase (PTK) Syk. To better understand the network, it is imperative to examine the roles of key signaling components like Syk and their most influential interactions. We will employ a computational approach to quantify the impact of Syk and other key enzymes and factors such as the effect of the amount of antigen on the B cell response.

Catalytically active Syk has been shown experimentally to play a central role in BCR signaling, but questions regarding its behavior still exist and the time frames in which critical interactions must occur have yet to be completely characterized. Experimentally, a mutated version of Syk called analog-sensitive Syk or Syk-AQL has been engineered to accept orthogonal inhibitors, i.e., inhibitors that have been synthesized to render the mutant kinase inactive almost immediately [1]. Furthermore, by replacing wild-type Syk with Syk-AQL creates B cells whose signaling capacity can be reduced or interrupted completely by the addition of the orthogonal inhibitor, experimentalists could then control the time that Syk remains active following receptor engagement, which helped to confirm how BCR signaling is modulated by the actions of Syk. Recently, a Syk-deficient B cell line was generated in which Syk-AQL expression can be induced in response to the drug tetracycline. Thus, in addition to being able to turn Syk off when desired, its expression level before activation can be adjusted if needed.

Computational modeling allows us to gain insight into Syk’s impact that was not previously possible with experimentation alone. We have developed a model built on a T cell receptor (TCR) signaling model originally created by Zheng *et al.* [2] and later expanded and used by Perley *et al.* [3] for cellular level control. Perley’s success in using Zheng’s model for prediction and open-loop control made it an ideal candidate to adapt for our B cell study. The signaling of B cell and of T cell can be divided into early interactions, which occur proximal to the membrane, and downstream interactions, which occur in the cytoplasm and ultimately lead to the nucleus. The dynamics of the downstream signaling are nearly identical between the cells, and thus this part of the Zheng model remained largely unchanged. The signaling dynamics of T cell and of B cell differ the most in their early signaling, which is where most model revisions were required.

In the past decade there have been a number of computational models, both stochastic and deterministic varieties, focusing on various aspects of B cell signaling, but none have considered impairment to Syk and the resulting effect on cell response. Stochastic simulations have been used by Tsourkas *et al.* [4] and Mukherjee *et al.* [5] while considering spatial dynamics of BCR signaling. The impact of affinity discrimination was considered by Tsourkas in their study, while Mukherjee investigated the roles of Syk and Lyn in immunoreceptor tyrosine-based activation motif (ITAM) phosphorylation. A deterministic model by Chaudhri *et al.* [6] considers a scope similar to Zheng’s T cell model, with the model covering both membrane proximal, early signaling events and downstream signaling events. This model pays particular interest to the role of phosphatases in the signal transduction. In 2012, Barua *et al.* [7] developed a deterministic model of B cell early signaling in order to study the feedback loops involving Lyn and how varying stimulation to the BCR leads to a range of dynamics in Syk. Impressively, the model incorporates every phosphorylation event for all six signaling components considered.

Our model is novel in its incorporation of Syk-AQL dynamics and given its scope, the inclusion of both early and downstream signaling, this allows us to investigate the impact of Syk modulation on a large number of signaling components. Instead of considering all possible phosphorylations for our 32 signaling components, our model considers only the most critical events in order to represent relevant physiological behavior and minimize model complexity. Understanding the means by which cell responses are determined is also of particular interest and the model will allow us to investigate the impact of both the amount of antigen and the level of Syk activity on the response. In this initial study we are particularly interested in the regulation of Erk and NF-*κ*B activity since both contribute to determining cell fate.

We will study how Erk and NF-*κ*B phosphorylation change with modulation of receptor affinity. Using parameter values derived largely by fitting the Perley model [3] to T cell data as nominal points, we use B cell data from Healy *et al.* [8] to then determine points in parameter space that allow us to reproduce data from cellular assays. Then, using the difference of Erkp, the sum of singly and doubly phosphorylated Erk, and NF-*κ*B as our metrics, we consider how cell response changes with receptor characteristics in both wild-type and mutant cells.

One interesting prediction of our model is that activation of Erkp and NF-*κ*B depend on ligand binding rates in a way that is nearly independent of the reverse rate for low values of the reverse rate and in a way that depends on a fixed ratio of powers of forward and reverse binding rates for higher values of the reverse binding rate. This is illustrated and discussed in Section 4.3 and is one indication that affinity (the ratio of forward and reverse binding rates) alone is not sufficient to characterize the response of a B cell to a given antibody.

The cell line used and experimental procedures are described in the experimental section. In the model section, we describe model construction and explicitly show equations and a diagram for the signaling dynamics. In the methods section, we discuss the sensitivity analysis used and criteria used to screen the parameter space. The discussion section includes biological background for the model and findings of the sensitivity analysis, parameter screening, and contour analysis. We also present a comparison of our model output with a dataset from Chaudhri *et al.* [6], and finish with some discussion of future direction and limitations.

## 2. Model Development

### 2.1. Biological Background

Since our B cell model is derived from an existing T cell model, we note here some of the primary components of B cell signaling, with a focus on aspects that are unique to B cells. Conventional T cells bind peptide antigens presented by major histocompatibility molecules whereas B cells can bind multiple molecular species through polymorphic cell surface immunoglobulins that serve as antigen receptors. The B cells work collaboratively with T cells to respond to monomeric antigens or independently of T cells to respond to polymeric antigens that cluster the BCR.

Once an antigen is bound and the BCR is aggregated, the signaling mechanisms at the B cell membrane are activated and an intricate system of molecular interactions initiates [9]. There are many kinases involved in this process; two connected with events proximal to the receptor in B cells are the Src-family PTK Lyn and the PTK Syk [10]. Syk plays a central role in the overall response of a B cell [11]. Unlike signaling in T cells, which depends on the Src-family PTK Lck to phosphorylate the first tyrosine of the ITAM of the TCR, the first ITAM tyrosine in B cells can be phosphorylated by Syk when Lyn, which is homologous to Lck in T cells, is not present [12]. Furthermore, if Syk is not expressed, BCR signaling cannot proceed. Following the initiation of BCR signaling, the regulation of BCR, Syk, and Lyn activity is orchestrated by feedback loops involving the aforementioned PTKs and a collection of regulatory enzymes.

The regulatory enzymes considered are the tyrosine-phosphatase SHP1, the C-terminal Src kinase Csk and its binding protein Cbp, and the phosphatase CD45 [13,14]. In addition to regulating fully activated Lyn along with CD45, SHP1 also dephosphorylates the ITAMs of the BCR and tyrosines Y342 and Y346 of Syk, thus reducing their activity. Note that SHP1 does not complete these actions until it has been activated itself by a Lyn, which has been dephosphorylated at its inhibitory site by CD45. After binding with a phosphorylated Cbp, activated Csk counteracts the dephosphorylation of Lyn promoted by CD45. The phosphorylation of Cbp is also promoted by CD45 activity. Gaining a better grasp of the timing of the interplay in these feedback loops is an important task as it provides insight into exactly how the BCR and primary PTKs are regulated and thus illuminates the overall sequence of events for signal transduction.

Once active, Syk phosphorylates several substrates and the resulting signals propagate into several downstream pathways that lead to the activation of downstream targets such as Erk, NFAT, and NF-*κ*B [9–11]. Following the translocation of these molecules into the nucleus, transcription begins and cell fate activation (proliferation), apoptosis (cell death), or anergy (chronic unresponsiveness) are determined. Interestingly, these cell responses have been found by Healy *et al*. [8] to correspond to characteristic combinations of the aforementioned targets. For example, anergic B cells exhibit signaling activity in the Erk and NFAT pathways, but not in the NF-*κ*B pathway [8]. Again, Erk and NF-*κ*B are of particular interest in this study due to their role in cell fate determination. The affinity of a receptor for an antibody $K_{\text{affinity}}=\frac{k_{\text{forward}}}{k_{\text{reverse}}}$ is a measure of how tightly a ligand binds to a receptor. However, for a given affinity, larger forward and reverse rates can allow the ligand to bind and unbind repeatedly and rapidly. Allowing this association and disassociation with the receptor to occur over prolonged periods of times and in the proper concentrations could be sufficient to simulate the low, chronic exposure of the BCR to antigen, which has been seen to induce anergy [15]. The causes for anergy have not been completely characterized, and we hope to use the model to gain a better understanding of the molecular triggers leading to anergy and the associated nonresponsiveness of B cells.

### 2.2. Model

**Figure 1:**
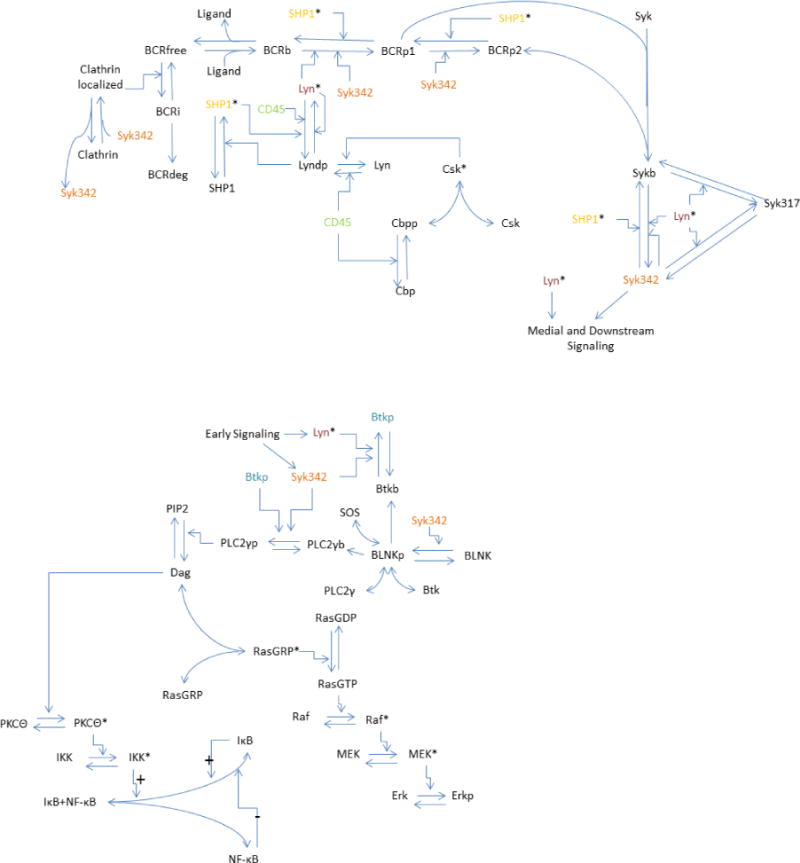
A depiction of the early, medial, and downstream signaling events induced by binding between B cell receptor and ligand, as described in the biological background section. Jagged arrows denote stimulations, curved arrows denote binding, straight arrows denote conversions, and color denotes species to appear repeatedly in the diagram. The plus and minus marks near the I*κ*B-NF-*κ*B disassociation reaction indicate which are positive feedbacks and which are negative feedbacks.

The model we present was developed based on the deterministic model for the TCR MAPK pathway created by Zheng [2] and extended by Perley [3]. Due to similarities in the signaling network, much of the model structure for the medial and downstream pathways required little modification. In particular, the model structure and equations for the MAPK pathway, which contains Erk, and the NF-*κ*B pathway, are analogous to those found in [16]. A diagram focusing on the signaling dynamics for our system is shown in Figure 1.

The model tracks the concentrations of 22 distinct species with the different forms of these species represented by individual variables. The model equations were formulated with mass action kinetics; conservation laws were used to reduce the number of variables in the system. The resulting model consists of 32 ordinary differential equations and has 114 parameters. Based on a sensitivity analysis described in the methods section, there are 12 kinetic parameters whose impact we will investigate in this study. Another important parameter is the ligand concentration, which is an external input to the model. We present equations for the revised early signaling dynamics only and refer to [3] for the remaining equations. We assume that there is no downstream activity prior to receptor stimulation; hence, our model is structured to be at zero at steady state for downstream variables like Erkp and NF-*κ*B. Additionally, we do not have any feedback between our downstream and upstream components, so setting these variables to zero is more of a baseline value than an absolute value.

### 2.3. Model Equations

#### BCR Activation

The receptor dynamics considered here include engagement of the BCR, ITAM tyrosine phosphorylation, Syk binding to the BCR, and BCR internalization, recycling and degradation. The key variables include free BCR *x_BCRfree_*, BCR bound by ligand *x_BCRb_*, singly-phosphorylated BCR *x_BCRp_*_1_, and doubly-phosphorylated BCR *x_BCRp2_*. The model formulation reflects how the kinase Syk can bind to either form of the phosphorylated BCR. Due to the positive promotion of ITAM tyrosine phosphorylation by membrane proximal PTKs [11], we assume that if Syk binds to a singly-phosphorylated BCR, that receptor will become doubly-phosphorylated before the kinase can unbind. Thus there is no term for unbinding from Sykb to BCRp1 in any of the model equations. Receptor internalization *x_BCRi_* is promoted by clathrin, which is localized $x_{{\mathrm{Clathrin}}_{\mathrm{local}}}$ to the membrane by Syk.

$$\begin{array}{l} \frac{{\mathrm{dx}}_{\mathrm{BCRfree}}}{\mathrm{dt}}=\left[\text{BCR recycling}\right]-\left[\text{BCR internalization}\right]\ldots \\ \;+\left[\text{Ligand disassociation}\right]-\left[\text{Ligand association}\right]\\ \;=r_{\mathrm{Recycling}}\cdot x_{\mathrm{BCRi}}-r_{\mathrm{Internalization}}\cdot x_{{\mathrm{Clathrin}}_{\mathrm{local}}}x_{{\mathrm{BCR}}_{\mathrm{Free}}}\ldots \\ \;+r_{\mathrm{disassociation}}\cdot x_{\mathrm{BCRb}}-r_{\mathrm{association}}\cdot \left[\text{Ligand}\right]\cdot x_{{\mathrm{BCR}}_{\mathrm{Free}}} \end{array}$$

$$\begin{array}{l} \frac{{\mathrm{dx}}_{\mathrm{BCRb}}}{\mathrm{dt}}=\left[\text{Ligand binding}\right]-\left[\text{Ligand disassociation}\right]\ldots \\ \;+\left[\text{ITAM}1\;\text{dephosphorylation}\right]-\left[\text{ITAM}1\;\text{phosphorylation}\right]\\ \;=r_{\mathrm{association}}\cdot \left[\text{Ligand}\right]\cdot x_{{\mathrm{BCR}}_{\mathrm{Free}}}-r_{\mathrm{disassociation}}\cdot x_{\mathrm{BCRb}}\ldots \\ \;+\left(r_{\mathrm{BCRp}1\mathrm{dephosphorylation}}\cdot x_{\mathrm{SHP}1*}+r_{\mathrm{BCRp}1\mathrm{dephosphorylation}\;\mathrm{by}\;\mathrm{phosphatases}}\right)x_{\mathrm{BCRp}1}\ldots \\ \;-\left(r_{\mathrm{BCRp}1\mathrm{phosphorylation}1}\cdot x_{\mathrm{Lyn}*}+r_{\mathrm{BCRp}1\mathrm{phosphorylation}2}\cdot x_{\mathrm{Syk}342}\right)x_{\mathrm{BCRb}} \end{array}$$

$$\begin{array}{l} \frac{{\mathrm{dx}}_{\mathrm{BCRp}1}}{\mathrm{dt}}=\left[\text{ITAM}1\;\text{phosphorylation}\right]-\left[\text{ITAM}1\;\text{dephosphorylation}\right]\ldots \\ \;+\left[\text{ITAM}2\;\text{dephosphorylation}\right]-\left[\text{ITAM}2\;\text{phosphorylation}\right]-\left[\text{Syk-BCR binding}1\right]\\ \;=\left(r_{\mathrm{BCRp}1\mathrm{phosphorylation}}\cdot x_{\mathrm{Lyn}*}+r_{\mathrm{BCRp}1\mathrm{phosphorylation}2}\cdot x_{\mathrm{Syk}342}\right)x_{\mathrm{BCRb}}\ldots \\ \;-\left(r_{\mathrm{BCRp}1\mathrm{dephosphorylation}}\cdot x_{\mathrm{SHP}1*}+r_{\mathrm{BCRp}1\mathrm{dephosphorylation}\;\mathrm{by}\;\mathrm{phosphatases}}\right)x_{\mathrm{BCRp}1}\ldots \\ \;+\left(r_{\mathrm{BCRp}2\mathrm{dephosphorylation}}\cdot x_{\mathrm{SHP}1*}+r_{\mathrm{BCRp}2\mathrm{dephosphorylation}\;\mathrm{by}\;\mathrm{phosphatases}}\right)\cdot x_{\mathrm{BCRp}2}\ldots \\ \;-\left(r_{\mathrm{BCRp}2\mathrm{phosphorylation}}\cdot x_{\mathrm{Syk}342}x_{\mathrm{BCRp}1}-r_{\mathrm{Syk}-\mathrm{BCR}\;\mathrm{binding}1}\cdot x_{\mathrm{Syk}}x_{\mathrm{BCRp}1}\right) \end{array}$$

$$\begin{array}{l} \frac{{\mathrm{dx}}_{\mathrm{BCRp}2}}{\mathrm{dt}}=[\text{ITAM}2\;\text{phosphorylation}]-[\text{ITAM}2\;\text{dephosphorylation}]\\ \;+\left[\text{Syk-BCR unbinding}1\right]-\left[\text{Syk-BCR binding}2\right]\\ \;=r_{\mathrm{BCRp}2\;\mathrm{phosphorylation}}\cdot x_{\mathrm{Syk}342}x_{\mathrm{BCRp}1}\ldots \\ \;-\left(r_{\mathrm{BCRp}2\mathrm{dephosphorylation}}\cdot x_{\mathrm{SHP}1*}+r_{\mathrm{BCRp}2\mathrm{dephosphorylation}\;\mathrm{by}\;\mathrm{phosphatases}}\right)\cdot x_{\mathrm{BCRp}2}\ldots \\ \;+r_{\mathrm{Syk}-\mathrm{BCR}\;\mathrm{unbinding}}\cdot x_{\mathrm{Syk}}-r_{\mathrm{Syk}-\mathrm{BCR}\;\mathrm{binding}2}\cdot x_{\mathrm{Syk}}x_{\mathrm{BCRp}2} \end{array}$$

$$\begin{array}{l} \frac{{\mathrm{dx}}_{\mathrm{BCRi}}}{\mathrm{dt}}=\left[\text{BCR internalization}\right]-\left[\text{BCR recycling}\right]-\left[\text{BCR degradation}\right]\\ \;=r_{\mathrm{Internalization}}\cdot x_{{\mathrm{Clathrin}}_{\mathrm{local}}}x_{{\mathrm{BCR}}_{\mathrm{Free}}}-r_{\mathrm{Recycling}}\cdot x_{\mathrm{BCRi}}-r_{\mathrm{Degradation}}\cdot x_{\mathrm{BCRi}} \end{array}$$

$$\begin{array}{l} \frac{{\mathrm{dx}}_{{\mathrm{Clathrin}}_{\mathrm{local}}}}{\mathrm{dt}}=\left[\text{Clathrin localization via Syk}342\right]-\left[\text{Clathrin delocalization}\right]\\ \;=r_{\text{Clathrin localization}}\cdot x_{\mathrm{Syk}342}x_{\text{Clathrin}}-r_{\text{Clathrin delocalization}}\cdot x_{{\mathrm{Clathrin}}_{\mathrm{local}}} \end{array}$$

#### Syk Activation

We consider four forms of Syk, three of which have been modified through binding or phosphorylation. The variable *x_Syk_* represents the amount of kinase that has not been activated and is unbound. The variable *x_Sykb_* is the basally active form of the kinase that has been bound to the BCR. The catalytically active form of Syk that has been phosphorylated at tyrosine Y342 and Y346 is denoted by *x_Syk_*_342_ and is responsible for enhancing signaling propagation. If either active form of the kinase becomes phosphorylated at tyrosine Y317 it is rendered inactive. This inactive form is denoted by *x_Syk_*_317_. The forms represented by *x_Syk_*_342_ and *x_Syk_*_317_ are still assumed to be bound to the BCR. As discussed below, each of these four forms of Syk can bind to an orthogonal inhibitor; this binding also renders Syk inactive.

$$\begin{array}{l} \frac{{\mathrm{dx}}_{\mathrm{Sykb}}}{\mathrm{dt}}=\left[\text{Syk-BCR binding}\right]-\left[\text{Syk-BCR unbinding}\right]\ldots \\ \;+\left[\text{Syk dephosphorylation at Y}342\right]-\left[\text{Syk phosphorylation at Y}342\right]\ldots \\ \;+\left[\text{Syk dephosphorylation at Y}317\right]-\left[\text{Syk phosphorylation at Y}317\right]\\ \;=(r_{\mathrm{Syk}-\text{BCR binding}1}\cdot x_{\mathrm{BCRp}1}+r_{\mathrm{Syk}-\mathrm{BCR}\;\mathrm{binding}2}\cdot x_{\mathrm{BCRp}2})x_{\mathrm{Syk}}-r_{\mathrm{Syk}-\mathrm{BCR}\;\mathrm{unbinding}}\cdot x_{\mathrm{Sykb}}\ldots \\ \;+\left(r_{\mathrm{Syk}342\;\mathrm{dephosphorylation}}\cdot x_{\mathrm{SHP}1*}+r_{\mathrm{Syk}342\;\mathrm{dephosphorylation}\;\mathrm{by}\;\mathrm{phosphatases}}\right)x_{\mathrm{Syk}342}\ldots \\ \;-\left(r_{\mathrm{Syk}342\;\mathrm{via}\;\mathrm{Lyn}*}\cdot x_{\mathrm{Lyn}*}+r_{\mathrm{Syk}342\;\mathrm{autophosphorylation}}\cdot x_{\mathrm{Syk}342}\right)x_{\mathrm{Sykb}}\ldots \\ \;+r_{\mathrm{Syk}317\;\mathrm{dephosphorylation}}\cdot x_{\mathrm{Syk}317}-r_{\mathrm{Syk}317\;\mathrm{phosphorylation}1}x_{\mathrm{Lyn}*}x_{\mathrm{Sykb}} \end{array}$$

$$\begin{array}{l} \frac{{\mathrm{dx}}_{\mathrm{Syk}342}}{\mathrm{dt}}=\left[\text{Syk phosphorylation at Y}342\right]-\left[\text{Syk dephosphorylation at Y}342\right]\ldots \\ \;+\left[\text{Syk dephosphorylation at Y}317\right]-\left[\text{Syk phosphorylation at Y}317\right]\\ \;=\left(r_{\mathrm{Syk}342\;\mathrm{via}\;\mathrm{Lyn}*}\cdot x_{\mathrm{Lyn}*}+r_{\mathrm{Syk}342\;\mathrm{autophosphorylation}}\cdot x_{\mathrm{Syk}342}\right)x_{\mathrm{Sykb}}\ldots \\ \;-\left(r_{\mathrm{Syk}342\;\mathrm{dephosphorylation}}\cdot x_{\mathrm{SHP}1*}+r_{\mathrm{Syk}342\;\mathrm{dephosphorylation}\;\mathrm{by}\;\mathrm{phosphatases}}\right)x_{\mathrm{Syk}342}\ldots \\ \;+r_{\mathrm{Syk}317\;\mathrm{dephosphorylation}}\cdot x_{\mathrm{Syk}317}-r_{\mathrm{Syk}317\;\mathrm{phosphorylation}2}\cdot x_{\mathrm{Lyn}*}x_{\mathrm{Syk}342} \end{array}$$

$$\begin{array}{l} \frac{{\mathrm{dx}}_{\mathrm{Syk}317}}{\mathrm{dt}}=\left[\text{Syk phosphorylation at Y}317\right]-\left[\text{Syk dephosphorylation at Y}317\right]\\ \;=\left(r_{\mathrm{Syk}317\;\mathrm{phosphorylation}}\cdot x_{\mathrm{Sykb}}+r_{\mathrm{Syk}317\;\mathrm{phosphorylation}2}\cdot x_{\mathrm{Syk}342}\right)x_{\mathrm{Lyn}*}\ldots \\ \;-2r_{\mathrm{Syk}317\;\mathrm{dephosphorylation}}\cdot x_{\mathrm{Syk}317} \end{array}$$

#### Syk-AQL dynamics

**Figure 2:**
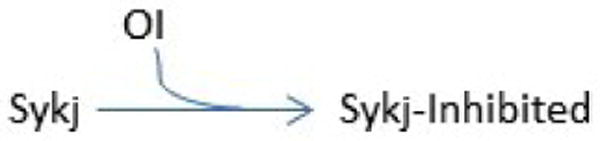
Orthogonal Inhibitor (OI) binding kinetics. Sykj denotes any of the modeled forms of Syk.

As discussed in the Introduction, Syk-AQL allows for Syk activity to be modulated through the addition of the orthogonal inhibitor (OI). The binding of the OI to the mutant Syk-AQL is also modeled using mass action kinetics and can be seen in Figure 2.

This binding results in the term $r_{\text{Inhibitor association}}\cdot [\mathrm{OI}]\cdot x_{{\mathrm{Syk}}_{j}}$ where *j* denotes any one of the forms of Syk modeled. These terms are subtracted from each of the equations for their respective forms of Syk. By conservation of mass, we have the following equation for orthogonally inhibited Syk:

$$\begin{array}{l} \frac{{\mathrm{dx}}_{\mathrm{Syk}-\mathrm{inh}}}{\mathrm{dt}}=\left[\text{Inhibitor association}\;-\;\text{Syk bound}\right]+\left[\text{Inhibitor association}\;-\;\text{Syk Y}342\right]\ldots \\ \;+[\text{Inhibitor association}\;-\;\text{Syk Y317}]+\left[\text{Inhibitor association}\;-\;\text{free Syk}\right]\\ \;=r_{\text{Inhibitor association}}\cdot \left[\mathrm{OI}\right]\cdot x_{\mathrm{Sykb}}+r_{\text{Inhibitor association}}\cdot \left[\mathrm{OI}\right]\cdot x_{\mathrm{Syk}342}\ldots \\ \;+r_{\text{Inhibitor association}}\cdot \left[\mathrm{OI}\right]\cdot x_{\mathrm{Syk}317}+r_{\text{Inhibitor association}}\cdot \left[\mathrm{OI}\right]\cdot x_{\mathrm{freeSyk}}. \end{array}$$

#### Lyn Activation

For the Src-family PTK Lyn (*x_Lyn_*) to become fully activated (x_Lyn*_), it must be dephosphorylated at Y508 (*x_Lyndp_*) and then go through an autophosphorylation reaction. We consider both events with the following equations:

$$\begin{array}{l} \frac{{\mathrm{dx}}_{\mathrm{Lyndp}}}{\mathrm{dt}}=\left[\text{Lyn dephosphorylation}\right]-\left[\text{Lyn phosphorylation}\right]\ldots \\ \;+\left[\text{Lyn de-autophosphorylation}\right]-\left[\text{Lyn autophosphorylation}\right]\\ \;=r_{\mathrm{Lyn}\;\mathrm{dephosphorylation}}x_{\mathrm{CD}45}x_{\mathrm{Lyn}}-r_{\mathrm{Lyn}\;\mathrm{phosphorylation}}x_{\mathrm{Csk}*}x_{\mathrm{Lyndp}}\ldots \\ \;+r_{\mathrm{Lyn}\;\mathrm{de-autophosphorylation}}x_{\mathrm{Lyn}*}-\left(r_{\mathrm{Lyn}*\;\mathrm{phosphorylation}}+r_{\mathrm{Lyn}*\;\mathrm{autophosphorylation}}x_{\mathrm{Lyn}*}\right)x_{\mathrm{Lyndp}} \end{array}$$

$$\begin{array}{l} \frac{{\mathrm{dx}}_{\mathrm{Lyn}*}}{\mathrm{dt}}=\left[\text{Lyn autophosphorylation}\right]-\left[\text{Lyn de-autophosphorylation}\right]\\ \;=\left(r_{\mathrm{Lyn}*\;\mathrm{phosphorylation}}+r_{\mathrm{Lyn}*\;\mathrm{autophosphorylation}}x_{\mathrm{Lyn}*}\right)x_{\mathrm{Lyndp}}\ldots \\ \;-\left(r_{\mathrm{Lyn}\;\mathrm{de-autophosphorylation}1}\cdot x_{\mathrm{CD}45}+r_{\mathrm{Lyn}\;\mathrm{de-autophosphorylation}2}\cdot x_{\mathrm{SHP}1*}\right)x_{\mathrm{Lyn}*}\ldots \\ \;-r_{\mathrm{Lyn}\;\mathrm{de}-\text{autophosphorylation by phosphatases}}\cdot x_{\mathrm{Lyn}*} \end{array}$$

#### Regulatory Enzyme Dynamics

Following the initiation of BCR signaling, the regulation of BCR, Syk, and Lyn activity is orchestrated by feedback loops involving the aforementioned PTKs and a collection of regulatory enzymes. The dynamic members of the regulatory subsystem are SHP1, Csk and Cbp, with their dynamics being driven by the amount of CD45. The activated forms of SHP1, Csk and Cbp are denoted by the variables *x_SHP_*_1*_, x_Csk__*_, and x_Cbpp__*_, respectively, and are modeled with the following equations:

$$\begin{array}{l} \frac{{\mathrm{dx}}_{\mathrm{SHP}1*}}{\mathrm{dt}}=\left[\text{SHP1 activation}\right]-\left[\text{SHP1 inactivation}\right]\\ \;=r_{\mathrm{SHP}1\;\mathrm{activation}}\cdot x_{\mathrm{Lyndp}}x_{\mathrm{SHP}1}-r_{\mathrm{SHP}1\;\mathrm{in}\mathrm{activation}}\cdot x_{\mathrm{SHP}1*} \end{array}$$

$$\begin{array}{l} \frac{{\mathrm{dx}}_{\mathrm{Csk}*}}{\mathrm{dt}}=\left[\text{Csk activation}\right]-\left[\text{Csk disassociation}\right]\\ \;=r_{\mathrm{Csk}\;\mathrm{activation}}\cdot x_{\mathrm{Cbpp}}x_{\mathrm{Csk}}-r_{\text{Csk disassociation}}\cdot x_{\mathrm{Csk}*} \end{array}$$

$$\begin{array}{l} \frac{{\mathrm{dx}}_{\mathrm{Cbpp}*}}{\mathrm{dt}}=\left[\text{Cbp phosohorylation}\right]-[\text{Cbp phosohorylation}]\\ \;=r_{\text{Cbp phosphorylation}}\cdot x_{\mathrm{Lyndp}}x_{\mathrm{Cbp}}-r_{\mathrm{Cbp}\;\mathrm{dephosphorylation}}\cdot x_{\mathrm{CD}45}x_{\mathrm{Cbpp}} \end{array}$$

#### Medial Signaling Dynamics (BLNK, BTK, PLC2γ)

The second messenger PLC2γ is critical for transducing a signal downstream following Syk activation. Before becoming fully activated, PLC2γ must bind to the linker protein BLNK and be phosphorylated by Syk and the Bruton’s tyrosine kinase (BTK). Here BTK must also bind to BLNK, and it is phosphorylated by Syk and Lyn before it becomes fully activated. These events are modeled by the following equations:

$$\begin{array}{l} \frac{{\mathrm{dx}}_{\mathrm{BLNKp}}}{\mathrm{dt}}=\left[\text{BLNK phosphorylation}\right]-\left[\text{BLNK dephosphorylation}\right]\\ \;=r_{\text{BLNK phosphorylation}}\cdot x_{\mathrm{Syk}342}x_{\mathrm{BLNK}}\ldots \\ \;-\left(r_{\mathrm{BLNK}\;\mathrm{dephosphorylation}}\cdot x_{\mathrm{SHP}1*}+r_{\text{BLNK dephosphorylation by phosphorylation}}\right)x_{\mathrm{BLNKp}} \end{array}$$

$$\begin{array}{l} \frac{{\mathrm{dx}}_{\mathrm{BTKb}}}{\mathrm{dt}}=\left[\text{BLNK-BTK binding}\right]-\left[\text{BLNK-BTK unbinding}\right]\\ \;=r_{\mathrm{BLNK}-\text{BTK binding}}\cdot x_{\mathrm{BTK}}x_{\mathrm{BLNKp}}-r_{\mathrm{BLNK}-\mathrm{BTK}\;\mathrm{unbinding}}\cdot x_{\mathrm{BTKb}} \end{array}$$

$$\begin{array}{l} \frac{{\mathrm{dx}}_{\mathrm{BTKp}}}{\mathrm{dt}}=\left[\text{BTK phosphorylation by Syk}342\right]+\left[\text{BTK phosphorylation by Lyn}*\right]\\ \;-\left[\text{BTK dephosphorylation}\right]\\ \;=\left(r_{\mathrm{BTK}\;\mathrm{phosphorylation}\;\mathrm{by}\;\mathrm{Syk}342}\cdot x_{\mathrm{Syk}342}+r_{\mathrm{BTK}\;\mathrm{phosphorylation}\;\mathrm{by}\;\mathrm{Lyn}*}\cdot x_{\mathrm{Lyn}*}\right)x_{\mathrm{BTKb}}\ldots \\ \;-r_{\mathrm{BTK}\;\mathrm{dephosphorylation}}\cdot x_{\mathrm{BTKp}} \end{array}$$

$$\begin{array}{l} \frac{{\mathrm{dx}}_{\mathrm{PLC}2\gamma b}}{\mathrm{dt}}=\left[\text{BLNK-PLC}2\gamma \;\text{binding}\right]-\left[\text{BLNK-PLC}2\gamma \;\text{unbinding}\right]\\ \;=r_{\mathrm{BLNK}-\mathrm{PLC}2\gamma \;\mathrm{binding}}\cdot x_{\mathrm{PLC}2\gamma }x_{\mathrm{BLNKp}}-r_{\mathrm{BLNK}-\mathrm{PLC}2\gamma \;\mathrm{unbinding}}\cdot x_{\mathrm{PLC}2\gamma b} \end{array}$$

dxPLC2γpdt=[PLC2γphosphorylation by Syk342]+[PLC2γphosphorylation by BTK∗]…−[PLC2γdephosphorylation]=(rPLC2γphosphorylationbySyk342⋅xSyk 342+rPLC2γphosphorylationbyBTK∗⋅xBTK∗)xBTKb…−rPLC2γdephosphorylation⋅xPLC2γp

Described in [3,16] are the remaining equations not shown here, *i.e.*, equations for Erkp, I*κ*B and NF-*κ*B, which are referenced below.

## 3. Materials and Methods

In this section we describe the experimental methods used to obtain Erk phosphorylation data and the algorithmic methods used for sensitivity analysis and parameter screening.

### 3.1. Experimental Protocols

#### 3.1.1. Cell Lines

Chicken DT40 B-cells lacking Syk were obtained from Dr. Tomohiro Kurosaki. Cells were cultured in RPMI 1640 media supplemented with 10% fetal calf serum, 1% chicken serum, 50 μM 2-mercaptoethanol, 1 mM sodium pyruvate, 100 IU/mL penicillin G, and 100 μg/mL streptomycin. Stable DT40 cell lines expressing analog sensitive Syk-AQL-EGFP (R428Q/M429L/M442A, referred to as Syk-AQL) were constructed using the Lenti-X Tet-On Advanced Inducible 105 Expression System (Clontech, Mountain View, CA, USA). To constitutively express the tetracycline-controlled transactivator, rtTA, in the Tet-On inducible system, the HEK293 cells were first infected with viral particles containing the pLVX Tet-On Advanced Regulator. Lentiviral particles were generated by co-transfecting HEK293T cells with 4 μg of pLVX-Tet-On, 4 μg of pHR’-CMV-∆R8.20 vpr, and 2 μg of pHR’-CMV-VSVG using Lipofectamine 2000 (Invitrogen, Carlsbad, CA, USA). The supernatants containing viral particles were harvested 48 h post-transfection and used to infect Syk-deficient DT40 cells. Two days after infection, cells were selected with 500 μg/mL G418 and screened for rtTA expression. Cells constitutively expressing rtTA protein were infected with lentiviral particles packaged with pLVX-Tight-Puro-Syk-AQL-EGFP as described above. After 48 h, cells were selected with 1 μg/mL puromycin and these cells were treated with 1 μg/mL doxycycline to induce Syk-AQL expression followed by screening for expression by Western blotting.

#### 3.1.2. Cellular Activation Assay

For the analysis of Erk phosphorylation, DT40 Syk-AQL-EGFP cells were treated with or without goat-anti-mouse IgM (10 (μg/mL) for the indicated periods of time at 37 °C and then lysed in buffer containing 25% sucrose, 2.5% SDS, 25 mM Tris/2.5 mM EDTA, 2.5 mg pyronin Y, and 2% 2-mercaptoethanol. The DNA in lysates was sheared by passing through a 26 G × 1/2 in needle. Proteins in the lysate were separated by SDS-PAGE, transferred to polyvinylidene difluoride membrane, and analyzed by Western blotting with anti-pERK (Cell Signaling p44/p42 MAPK (T202/Y204) rabbit 4370S), and anti-Syk (Santa Cruz N-19 rabbit) antibodies. The results of these assays were used for parameter screening and will be referenced in the results section.

### 3.2. Sensitivity Analysis

Our first objective was to identify parameters that produce behavior that fits B cell data. This is important as our nominal parameters largely came from parameters estimated by fitting to T cell data. In order to obtain a computationally tractable search, we first conducted a sensitivity analysis to identify the parameters with the greatest influence on model output associated with our available data.

We were concerned with fitting the model output to Erkp and I*κ*B data reported by Healy *et al.* in [8], Erkp data obtained as described in the previous section, and NF-*κ*B data reported by Oh *et al.* in [1]. Recall that Erkp denotes the sum of singly and doubly phosphorylated Erk. The experimental conditions we sought to simulate were administrations of ligand at time *t* = 0 in doses ranging from 5.5 to 150 (μg/mL. The data from Healy *et al.* was used for model fitting through parameter screening, and so the form of this data was taken into consideration during sensitivity analysis. The measurements taken by Healy *et al.* were reported relative to the basal or unstimulated phosphorylation of a species. Thus, to evaluate the fitness of a set of parameters, we ran the model to steady state and recorded the value of $x_{\mathrm{out}}^{\mathrm{Basal}}$, where *out* = *Erkp* or I*κ*B throughout all subsequent sections, before continuing the simulation with the addition of ligand.

Fitness was quantified using the objective

(1) $$J_{\mathrm{out}}=\left(y_{\mathrm{obs}}-y_{\mathrm{simulated}}\right)/\sigma_{\mathrm{out}}$$

Given that the basal and simulated values both depend on parameters, for our initial sensitivity analysis we chose to compute the sensitivity of

(2) $$y_{\mathrm{sim}}=\frac{x_{\mathrm{out}}^{\mathrm{Stimulation}}(t_{\mathrm{obs}},p_{k})}{x_{\mathrm{out}}^{\mathrm{Basal}}(p_{k})}$$

with respect to variations in parameters *p_k_*. Note that *p_k_* is the *k^th^* point in our parameter screening. To estimate the uncertainty in the data *σ_out_* for Equation (1), we assumed a linear dependence of *σ_out_* on *y_obs_* and conducted a linear regression using the information in Figure 2C of [8]. We found that the error in the measurements could be reasonably approximated by

(3) $$\sigma_{\mathrm{out}}=0.0127+0.3084\cdot y_{\mathrm{obs}}$$

where *y_obs_* is an observed measurement. Given the total number of model parameters and the cost associated with varying them, we partitioned parameters into seven distinct groups and conducted a sensitivity analysis with respect to each group when determining which parameters to screen initially. These groups of parameters were detrained using natural divisions such as BCR dynamics, Syk dynamics, regulatory enzyme and Lyn dynamics, Erk pathway dynamics, *etc*. A study by Zheng [2] comparing local derivative-based sensitivity methods and global variance-based methods found that global parameter sensitivities were necessary to capture model behavior when considering a large parameter space, but that there were no significant difference between Sobol analysis and the other variance based methods considered. Given the relative independence of these groups, we calculated only primary Sobol sensitivities [17] to estimate the sensitivity of the outputs, normalized as in Equation (2), to each specified parameter. The sensitivity, $S_{p_{k}}(t)=\frac{{\mathrm{Var}}_{p_{k}}\left(E_{p\neq p_{k}}\left[x_{\mathrm{out}}^{\mathrm{Stimulation}}|p_{k}\right]\right)}{\mathrm{Var}\left(x_{\mathrm{out}}^{\mathrm{Stimulation}}\right)}$, for a given parameter was computed at integer values *t* = 0, …, 30 using the method based on sparse-grid interpolation as described in [18]. This expression is designed to capture the relative sensitivity of the output as a function of one particular parameter *p_k_*, averaged over the other parameters. That is, if we fix *p_k_*, we can determine the average behavior as we vary the remaining parameters, and then determine how this average changes as a function of *p_k_*. These calculations were carried out in log space in each parameter, with a range of one order of magnitude above and below the nominal value for each parameter.

To match the conditions in much of that in [8], we used 20 (μg/mL for the stimulation amount at time *t* = 0. For each parameter considered, we calculated the median of the sensitivities for that parameter over the times considered. The value 0.15 was found to be a natural threshold for each group, and if the median was less than 0.15, we concluded the parameter was insensitive and excluded it from future parameter screening. This criterion left us with 12 parameters to consider for the parameter screening. Plots of these sensitivity values are included in Supplementary Materials.

### 3.3. Parameter Screening

Using Latin Hypercube Sampling (LHS), we screened parameter space for points that we consider acceptable if they produce simulations satisfying |*J_out_*| ≤ *η* or equivalently

(4) $$y_{\mathrm{obs}}-\eta \sigma \leq y_{\mathrm{sim}}\leq y_{\mathrm{obs}}+\eta \sigma$$

The first screening used the following data from Healy *et al.*: an Erk measurement at time *t* = 5 minutes and dose responses for I*κ*B all measured at time *t* = 15 minutes. The doses provided in the dose response experiment were 5.5, 16.5, 50, and 150 μg/mL.

The second screening was with respect to our data and also used the condition Equation (4) to determine acceptability. However, in the acceptability condition for this screening, we modified the calculation of *y_sim_* in that we calculate phosphorylation relative to the ending value rather than the basal value. That is, the signal intensities for the Western blots from our data were normalized by their ending phosphorylation levels to avoid the magnification of errors that would result from a small initial value. Applying the same normalization procedure to simulated data gives the form

(5) $$y_{\mathrm{sim}}=\frac{x_{\mathrm{out}}^{\mathrm{Stimulation}}(t_{\mathrm{obs}},p_{k})}{x_{\mathrm{out}}^{\mathrm{Stimulation}}(t_{\mathrm{final}},p_{k})}$$

In this case the uncertainty in our experimental measurements was determined by calculating the standard deviation of the three replicates.

The data from Oh *et al.* [1] consisted of data for wild-type and mutant B cells, which featured Syk-AQL. The mutant data was reported relative to wild-type activity. The wild-type data was used to ensure that we achieved reasonable behavior in NF-*κ*B after using data from Healy *et al.* to fit I*κ*B, the model variable that directly preceded NF-*κ*B. Using sensitive parameters relating to Syk dynamics as a guide to select a small subset of parameters to tune manually, the mutant data was used to determine a separate parameter set to reproduces this mutant (Syk-AQL) behavior.

### 3.4. Contour Analysis

In order to investigate the dependence of Erk and NF-*κ*B activation on ligand-receptor binding rates, we simulated the model at a dose of 20 (μg/mL anti-BCR over a product grid of forward and reverse binding rates. This was done for each of wild-type, mutant with no orthogonal inhibitor, and mutant with a dose of 1 μM orthogonal inhibitor. The parameter grid was constructed using evenly spaced points in log scale over ranges for forward and reverse binding rates found in literature [4,6].

In order to avoid numerical inaccuracies associated with overly stiff parameters, we halted any simulation that took longer than fifteen minutes during the contour analysis. For these grid points, we used the built-in MATLAB function *griddata* in order to interpolate the corresponding values.

The parameter screening, sensitivity analysis, and contour analysis scripts were implemented in MATLAB 2012a. The script was parallelized to run on a Sun Server X3–2 server with two Intel Xeon E5–2690 processors and 160 GB RAM. For the parameter screening and sensitivity analyses, parameter ranges were set to two orders of magnitude on either side of the nominal parameters for each group except the fourth group (see Section 4.2 and Table 1).

## 4. Results

### 4.1. Sensitive Parameters

Primary Sobol sensitivities [17] were calculated for each output and we analyzed the distribution of the sensitivities over time. The following parameters were considered during the parameter screening. Inclusion in the parameter screening meant that the parameter did not violate the criterion that median *S*(*t*) < 0.15. The box and whisker plots for parameter sensitivities leading to this criterion are included in the supplementary information Figure S1. We ultimately sought for the model to produce a graded response to increasing dosages of anti-BCR stimulation in Syk342 and then let that gradation propagate downstream; thus, the results of the sensitivity analysis match what one would expect as they correspond to key signaling reactions.

The parameters in group one correspond to BCR dynamics, and *rw*0*_kf_* specifically represents the forward rate of the ligand binding reaction to the BCR. Group two contains parameters related to Syk activation, and *rw*7*_kr_* is the reverse rate of the phosphorylation reaction for the Y342 tyrosine on Syk. Parameter *rw*9*_kf_* is the forward rate in the phosphorylation reaction for the Y317 tyrosine on Syk that has already been phosphorylated at Y342. Group three is comprised of parameters from the regulatory enzyme subsystem. A sensitivity analysis was not conducted with respect to this group due to issues with stiffness. We describe next steps to examine this stiffness and future plans to expand the regulatory enzyme subsystem to become fully dynamic in Section 5.

Groups four and five consisted of parameters relating to rates for reactions involving medial signaling components BLNK, PLC*γ*, Bruton’s Tyrosine Kinase (BTK). For group four, parameter *rw*15*_kf_* is the rate at which BLNK is phosphorylated by Syk342, while *rw*16*_kf_* and *rw*16*_kr_* are the forward and backward rates for the binding of PLC*γ* to the linker protein BLNK. In group five, *r*12*s_kf_* is the forward rate at which Syk342 phosphorylates bound PLC*γ*. Parameters r13*_kf_* and *r*13*k_r_* represent the rate at which PLC*γ* phosphorylates the phospholipid PIP2 and the corresponding rate of dephosphorylation. Finally, group six is made up of medial signaling parameters for reactions involving the kinase PKC and also the downstream MAPK pathway leading to Erk. Parameter *r*18*_kf_* is the rate at which Erk is phosphorylated by MEK. Parameter *r*19*_kf_* is the rate at which the enzyme SOS binds to phosphorylated BLNK. Finally, Group seven consists of parameters for reactions related to the NF*κ*B pathways. Here *r*38*_kf_* is the rate of phosphorylation of I*κ*B by the kinase IKK.

### 4.2. Parameter Screening and Fitting

To find a set of parameters that qualitatively match a variety of data, we first screened with respect to the data from Healy *et al.* [8] and required |*JErkp*| ≤ 1 and |*J*_I*κ*B(*dose1*)_| ≤ 2. We found seven parameter vectors that met the criteria among the 1800 candidates considered. Due to large per-simulation time requirements, large objective values for doses #3 and #4 of the dose response experiments, and tradeoffs between Erk costs and I*κ*B costs, we determined we would need to manually tune parameters related to I*κ*B to achieve reasonable fits at all four doses.

**Figure 3:**
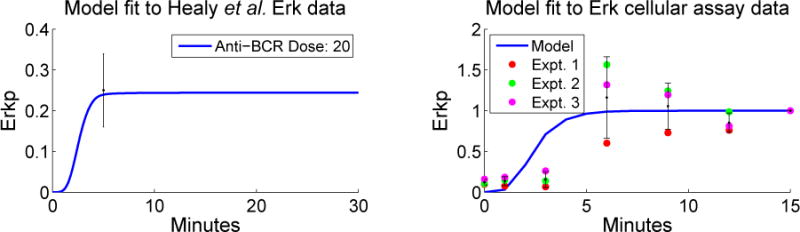
Simulations using $p_{\mathrm{WT}}^{*}$ compared with experimental data. On the left, a simulation for the Erkp time course (normalized by total Erk) with 20 μg/mL anti-BCR is shown with the mean from Healy *et al.* [8] at time *t* = 5 and one standard deviation interval of uncertainty. On the right, simulations using $p_{\mathrm{WT}}^{*}$ and 10 μg/mL anti-BCR (normalized by Erk at time *t* = 15) are compared with Erkp triplicate data from Section 3.1.2.

We next screened the seven accepted parameter vectors for fitness to the Erkp data obtained as in Section 3.1.2 and normalized as in Equation (5) with a threshold of *η* = 1. From this screening, we selected one parameter vector *p* based upon the smoothness of its Erkp time course, time to full Erkp activation, agreement with intermediate Erkp data points, and smoothness of non-degraded I*κ*B time courses. Simulations using *p* are shown in Figure 3. For this Erkp data, we did not do any local optimization, but rather focused on the qualitative response. The right panel in Figure 3 indicates both the variability in experimental data and good qualitative agreement between these data and simulation.

**Figure 4:**
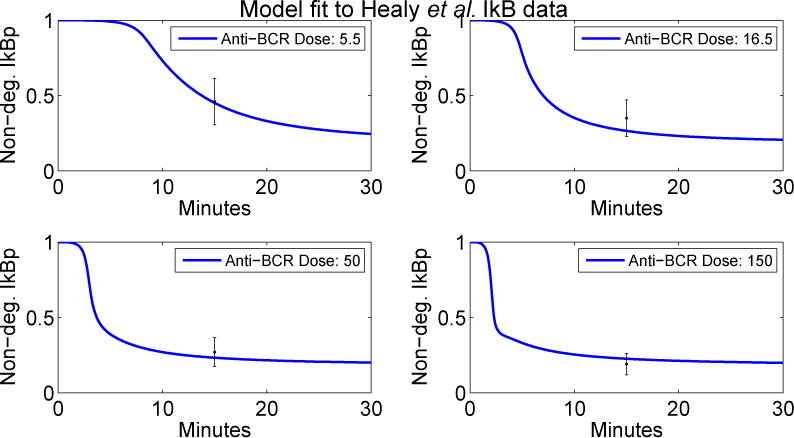
Simulations using $p_{\mathrm{WT}}^{*}$ compared with I*κ*B data from Healy *et al.* [8]. Simulations for non-degraded I*κ*B (normalized by total I*κ*B) are shown (left to right, top to bottom) for 5.5, 16.5, 50 and 150 (μg/mL anti-BCR, with all measurements taken at time *t* = 15 and one standard deviation interval of uncertainty.

**Figure 5:**
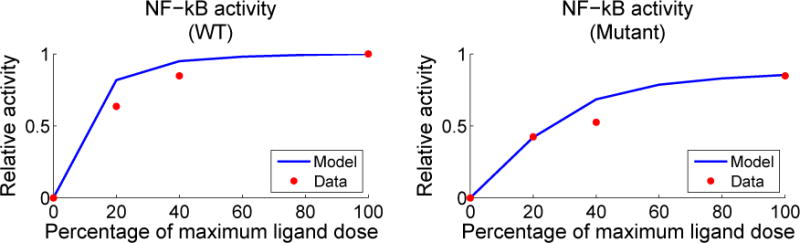
Anti-BCR dose response curves compared with experimental data from Oh *et al.* [1]. On the left, a simulation using $p_{\mathrm{WT}}^{*}$ (normalized by WT activity at the maximum dose) is shown to qualitatively agree with the wild-type NF-*κ*B data ( 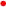). On the right, a simulation with the parameter vector $p_{\mathrm{Mutant}}^{*}$ (also normalized by WT activity at the maximum dose) is shown with NF-*κ*B data ( 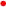) from B cells with Syk-AQL activity.

From the vector *p*, we improved our fits for I*κ*B by manually tuning parameters in the I*κ*B pathway. The parameters that were adjusted were the rate of I*κ*B production and the rate of NF-*κ*B production. This final manual tuning led us to the parameter vector we call $p_{\mathrm{WT}}^{*}$. The final fits for I*κ*B can be seen in Figure 4. We were able to achieve qualitative agreement to the wild-type NF-*κ*B data of Oh *et al.* [1] without any further changes to parameters, as seen in the left panel of Figure 5.

Since mutant Syk-AQL has experimentally different NF-*κ*B response compared with WT, we manually tuned the sensitive parameters associated with Syk dynamics. We found that increasing the rate of Y317 phosphorylation *rw*9*_kf_* allowed us to fit two of the three nonzero data points. The agreement to the mutant data with this new parameter vector $p_{\mathrm{Mutant}}^{*}$ can be seen in the right panel of Figure 5. Intuitively, this corresponds to inhibiting a larger fraction of Syk, and thus there is less Syk available to propagate a signal. Interestingly, we could also achieve the same fits to mutant data by lowering the total amount of Syk in the cell. This was reminiscent of the effects of the drug tetracycline, which can regulate the amount of kinase prior to stimulation. Note that the measurements used from Oh *et al.* were reported relative to phosphorylation levels observed following an experiment where cells were stimulated using PMA and ionomyocin. We do not simulate the effects of ionomyocin in this work since calcium is not modeled, so our simulated activity in the right panel of Figure 5 is relative to the final phosphorylation level observed in simulated wild-type activity.

**Figure 6:**
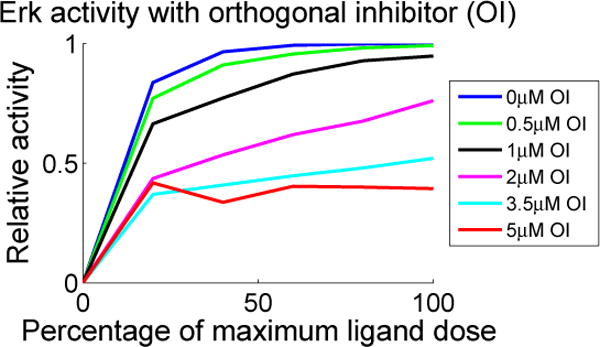
Anti-BCR dose response curves for baseline Syk-AQL activity and inhibited activities. The curves show simulated relative activity for Erkp measured at *t* = 5 after applying ligand and orthogonal inhibitor (μM) simultaneously All curves have been normalized by Erk activity at the maximum dose with no orthogonal inhibitor added. The color of the curve corresponds to the amount of orthogonal inhibitor specified in the legend.

In Figure 6, we plot predicted dose response curves associated with the parameter vector $p_{\mathrm{Mutant}}^{*}$ as a function of ligand dose, one curve for each of several doses of orthogonal inhibitor (the OI doses are specified in μM in the legend). The simulation values are given at *t* = 5 minutes. To investigate the qualitative response, we express the ligand dose in each case as a percentage of saturating dose. As seen in Figure 6, our model exhibits a clear dose response to antigen. Additionally, it is clear in the figure that the orthogonal inhibitor limits the Erkp response; activity level is reduced as the amount of inhibitor increases, suggesting that active Syk is critical to propagate the signal and may be a limiting quantity.

### 4.3. Contour Analysis

As shown by Healy *et al.* in [8], there is full signaling activity through the Erk pathway and limited activity in the NF-*κ*B pathway during an anergic response. To investigate a variety of affinities that could induce anergy, we vary the forward and reverse kinetic rates for BCR binding and consider the cell activity as a function of the binding rates. We seek to find areas of the grid of binding rates that lead to high Erkp activity and low NF-*κ*B activity. We have constructed contour plots for normalized Erkp activity minus normalized NF-*κ*B activity for several scenarios: WT B cells, mutant B cells without OI added, mutant B cells with 1 μM of OI added. The contour plots allow us to ascertain relationships between the binding rates associated with the responses we found.

**Figure 7:**
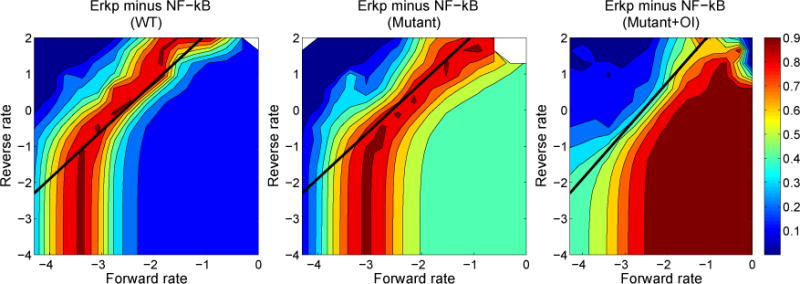
Contour plots for wild-type (WT), mutant without orthogonal inhibitor and mutant with 1 μM orthogonal inhibitor. The diagonal black line has a slope equal to 4/3. Regions with high values correspond to large Erkp response and small NF-*κ*B response (both responses normalized by their maximum WT activity), and hence possible regions of anergy. Both rates are shown in log scale.

As seen in Figure 7, for each scenario the response at low values of the reverse binding rate is qualitatively different from the response at higher values of reverse binding rate. At low values, the response depends only on the forward binding rate, while at higher values the response depends more or less linearly in log space on both binding rates. The slope for this linear relationship is not 1, however, which would be the case if the response depended on the standard affinity, $K_{a}=\frac{\mathrm{rw}0_{\mathrm{kf}}}{\mathrm{rw}0_{\mathrm{kr}}}$. As seen in the contour plots, the response above the value *rw*0*_kr_* > −0.5 is reasonably described as a function of l*og rw*0*_kf_* − α log *rw*0*_kr_*. This leads to a kind of power law affinity, $K_{a,\alpha }=\frac{\mathrm{rw}0_{\mathrm{kf}}}{{\left(\mathrm{rw}0_{\mathrm{kr}}\right)}^{\alpha }}$, where the multiplier *α* = 3/4 is the reciprocal of the slope of the linear relationship in the contour plot. The origin of the power law affinity will be investigated in future analysis of the dynamical system.

**Figure 8:**
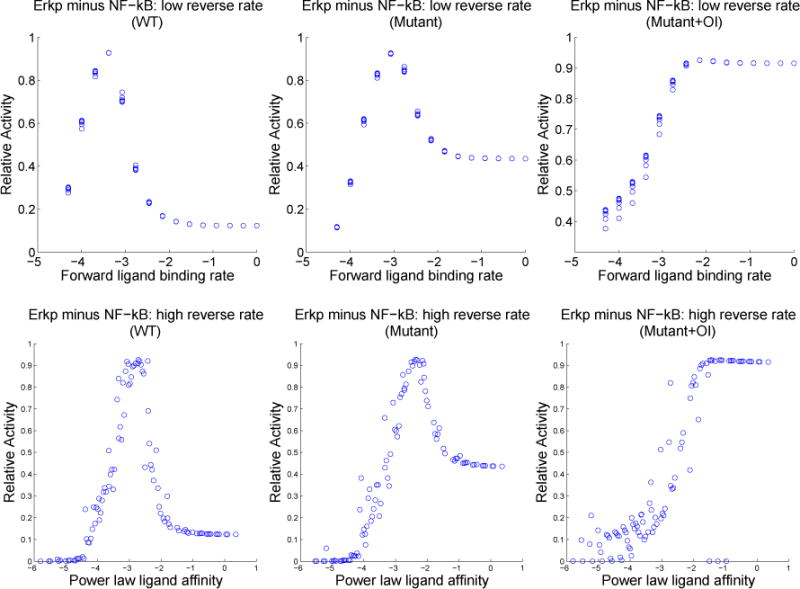
Plots of normalized Erkp minus normalized NF-*κ*B (each normalized by their maximum WT activity) over a product grid of forward and reverse binding rates as in the contour plots above, but separated into regions of high and low reverse rates. The first column is wild-type simulation, the second column is mutant simulation without orthogonal inhibitor, and the third column is mutant simulation with 1 μM orthogonal inhibitor. Rates are shown in log scale.

To illustrate these dependencies, we plot in Figure 8 the responses in the low reverse rate region against the forward rate *rw*0*_kf_* and the responses in the high reverse rate region against the power law affinity. As expected from the contour plots, the plots in Figure 8 show a clear dependence on forward rate alone in the region of low reverse rate and a reasonably clear dependence on the power law affinity in the region of high reverse rate.

There are higher plateaus of Erkp-NF-*κ*B present in the mutant plots (middle and right) of Figure 8. Plots of each quantity separately (not shown) demonstrate that plateau levels of Erkp are relatively unchanged while NF-*κ*B is suppressed in these mutants. These higher plateaus lead to the question of whether it is easier to induce and observe anergy in B cells with Syk-AQL than in WT. If so, this could have important implications for attempts to produce mice with these mutant B cells.

In order to further understand the effect of Syk-AQL and OI on Syk, we consider the allocation of Syk in each scenario. Using the power law affinity, we find that the variables *x_Sykb_*, *x_Syk_*_342_, and x_317_ all follow a sigmoidal course. Note that a percentage of total Syk is also allocated to other variables, such as free, unbound Syk, and to Syk bound to clathrin; since our focus is on the active forms of Syk, we omit these other forms. We find that Syk-AQL with no orthogonal inhibitor mimics fairly closely the response of wild-type, except that Syk342 is somewhat reduced. As expected, Syk-AQL with orthogonal inhibitor shows a marked decrease in these three forms of Syk, with the balance migrating to inhibited Syk.

The analysis in this section has several possible biological implications. The moderate reduction in Syk-AQL activity compared with wild-type suggests that the level of NF-*κ*B activation, which is reduced in Syk-AQL cells relative to wild-type, is more sensitive to small reductions in kinase activity than Erkp activation, which is essentially unchanged. Since this profile of high Erkp and reduced NF-*κ*B is consistent with anergy, this implies that anergy is particularly sensitive to small changes in catalytic activity. A second possible implication derives from the observed dependence of Erkp and NF-*κ*B on the power law affinity. This implies that B cells may respond to certain details of binding and unbinding rates for ligands rather than simple affinity alone. These observations provide a platform upon which to plan future experimental approaches and to predict experimental outcomes to further evaluate the role of Syk and changes in its catalytic activity in determining cell fate decisions following BCR engagement.

### 4.4. Independent Dataset Comparison

**Figure 10:**
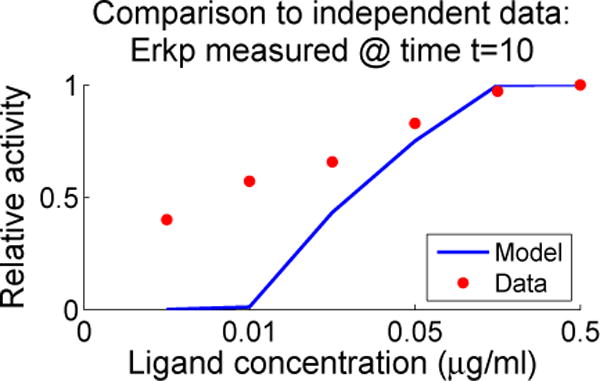
Anti-BCR dose response curves resulting from $p_{\mathrm{WT}}^{*}$; the figure shows ligand dose response for Erkp resulting from $p_{\mathrm{WT}}^{*}$ as compared with data from Chaudhri *et al*. [6]. As with the data, the simulation curve is normalized by the simulated value at the maximum dose 0.5 μg/mL.

We compared the model to an independent dataset from Chaudhri *et al*. [6]. These data were not used in screening the parameters; the comparison is presented in Figure 10. The Chaudhri data include ligand concentrations that are much smaller than those available in our training data and indicate a relatively large activation even at very small ligand concentrations. Our model displays significantly smaller activity levels than those seen in the Chaudhri data at these low ligand concentrations. We believe that further parameter screening could produce better agreement to these data, but the underlying question is somewhat deeper in view of the phenomenon of anergy, in which B cells display reduced response to higher levels of ligand concentration. Experiments have shown that a low constant signal [15] can drive a B cell to become anergic and thus relatively unresponsive to the presence of antigen. Hence the question is not only what is the effective level of phosphorylation of Erk at low doses of ligand but also what is the effect of such low doses over extended periods of time. This is consistent with our model predictions of relatively high levels of Erkp activity and low levels of NF-*κ*B activity in response to small amounts of active Syk. However, our model also suggests that the details of forward and reverse binding rates may also play a role in anergy.

## 5. Conclusions and Future Directions

We have constructed a deterministic model of B cell signaling, with a focus on the role of Syk in modulating the activity of Erk and NF-*κ*B. In particular, we include dynamics for the mutant kinase Syk-AQL, which experimentally displays dynamics that are qualitatively similar to wild-type dynamics in the absence of orthogonal inhibitor but can be modulated through the addition of orthogonal inhibitor. With the correct choice of parameters, our model reproduces data from recent cellular assays and qualitatively matches trends from datasets in the literature.

We sought to explore the kinetic rate constants associated with ligand binding that produced high relative activation of Erkp and low relative activation of NF-*κ*B. These signaling conditions have been previously associated with anergy. We found that at different levels of *rw*0*_kr_* our responses actually depended on quantities other than the standard affinity constant. For low levels of *rw*0*_kr_*, the model predicts that the response depends only on the forward rate of BCR binding *rw*0*_kf_*. At higher levels of *rw*0*_kr_*, the model predicts that the response depends on a power law form of the affinity constant, $K_{a,\alpha }=\frac{\mathrm{rw}0_{\mathrm{kf}}}{{\left(\mathrm{rw}0_{\mathrm{kr}}\right)}^{\alpha }}$. These predictions were robust for WT and mutant simulations. Given the complexity of the dynamical system, a model reduction will likely be necessary in order to analytically investigate the origin of the power law affinity underlying the model response.

Insight into the model prediction that NF-*k*B is more sensitive than Erkp to changes in signaling activity is found when considering the relative amplification in each pathway. For both Erkp and NF-*κ*B, we considered the relative change in response between wild-type and mutant with orthogonal inhibitor simulations. The relative changes were both with respect to the signaling component DAG, the last signaling component to influence both pathways. We calculated the difference between Erkp in wild-type and mutant+OI simulations and then divided by wild-type Erkp simulation to get the normalized change in Erkp. We made a similar calculation using DAG, normalized by wild-type DAG simulation, and then took the ratio of the normalized change in Erkp to the normalized change in DAG. This gives us a measure of the amplification of the DAG signal in the response of Erkp. We likewise calculated the simulated amplification of DAG in the response of NF-*κ*B. We found the amplification for Erkp to be ≈0.28 and the amplification for NF-*κ*B to be ≈0.99. That is, the response of NF-*κ*B to DAG is nearly 1:1, while the response of Erkp to DAG is reduced to roughly one-fourth of the incoming signal. These estimates agree with the findings in contour analysis that if there is a reduction in signaling activity to Syk, and thus DAG, then NF-*κ*B will be more affected than Erkp. The mechanisms and parameters in these two pathways are structurally distinct: the Erkp pathway is based on mass-action kinetics, while the NF-*κ*B pathway includes promotion of PKCΘ* by DAG and a feedback loop involving NF-*κ*B. Further experiments are needed to validate these predictions. One approach to this might be to use the DAG analog PMA as a means of effectively altering the level of DAG and investigate the resulting changes in Erkp and NF-*κ*B experimentally.

Planned expansions to the model include stimulation by ionomycin, the addition of Ca^2+^ dynamics, and the addition of the NFAT pathway. We plan also to restructure the dynamics of CD45, which is constant in the current version of the model; this modification will impact the regulatory enzyme dynamics as they are driven by CD45 activity.

One of the difficulties with this model is the stiffness of the differential equations; for a large subset of parameter space the model takes one to tens of minutes for a simulation of 30 minutes. This stiffness limits our ability to explore the parameter space fully. Model stiffness prevented sensitivity analysis with respect to the parameters for regulatory enzyme dynamics, which made up group three of Table 1. Stiffness also presented issues during other sensitivity analysis trials and during the parameter screening and so we will seek to address this issue in future studies. We believe the improvements to CD45 dynamics will alleviate at least some of the issues with stiffness.

As seen in the right panel of Figure 6, there is a discrepancy between our model and the activity observed by Chaudhri *et al.* [6]. It is not clear whether this limitation can be resolved via the tuning of ligand binding parameters or if there are additional mechanisms needed to capture the response to lower levels of ligand.

In general, the ways in which the modulation of Syk changes the response of Erk, NFAT, and NF-*κ*B is an important question of interest for our group. Our model is an early attempt to disentangle the behavior of Syk from these downstream responses. While there is much left to be improved in our model, we believe that it will be an important tool in our search to understand the mechanisms underlying the onset of anergy in B cells. Beyond that, we believe that our model may be used as in [3] as the basis for model-informed control strategies to achieve desired cellular responses.

## Supplementary Material

Figure S1

## Figures and Tables

**Figure 9:**
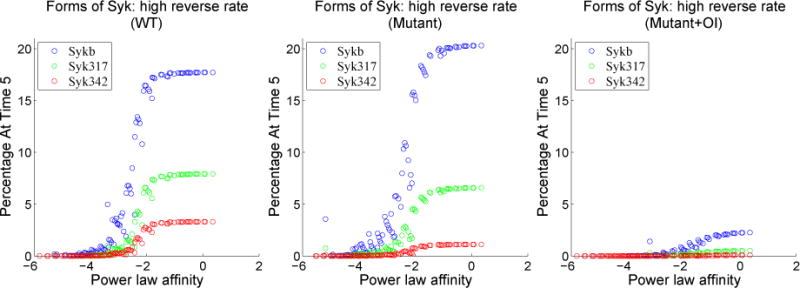
Plots for three forms of Syk in the model as a function of the power law affinity constant for wild-type and mutant behavior. We notice lower phosphorylation levels at both tyrosine Y317 and Y342 in the mutant cells. After the addition of 1 μM orthogonal inhibitor to the mutant cell there is the expected decrease in overall activity; the balance is accounted for by inactive forms of Syk.

**Table 1:** Sensitive parameters.

	Reactions	Parameters
Group 1	BCR dynamics	*rw*0*_kf_*
Group 2	Syk activation	*rw*7*_kr_*, *rw*9*_kf_*
Group 3	Regulatory enzyme dynamics	N/A
Group 4	Medial signaling	*rw*15*_kf_*, *rw*16*_kf_*, *rw*16*_kr_*
Group 5	Medial signaling	*r*12s*_kf_*, *r*13*_kf_*, *r*13*_kr_*
Group 6	Erk pathway dynamics	*r*18*_kf_*, *r*19*_kf_*
Group 7	NF-*κ*B pathway dynamics	*r*38*_kf_*

## References

[1] Oh H, Ozkirimli E, Shah K, Harrison ML, Geahlen RL (2007). Generation of an Analog-sensitive Syk Tyrosine Kinase for the Study of Signaling Dynamics from the B Cell Antigen Receptor. J Biol Chem.

[2] Zheng Y, Rundell A (2006). Comparative study of parameter sensitivity analyses of the TCR-activated Erk-MAPK signalling pathway. IEE Proc Syst Biol.

[3] Perley JP, Mikolajczak J, Harrison ML, Buzzard GT, Rundell AE (2014). Multiple Model-Informed Open-Loop Control of Uncertain Intracellular Signaling Dynamics. PLoS Comput Biol.

[4] Tsourkas PK, Somkanya CD, Yu-Yang P, Liu W, Pierce SK, Raychaudhuri S (2012). Formation of BCR oligomers provides a mechanism for B cell affinity discrimination. J Theor Biol.

[5] Mukherjee S, Zhu J, Zikherman J, Parameswaran R, Kadlecek TA, Wang Q, Au-Yeung B, Ploegh H, Kuriyan J, Das J (2013). Monovalent and Multivalent Ligation of the B Cell Receptor Exhibit Differential Dependence upon Syk and Src Family Kinases. Sci Signal.

[6] Chaudhri VK, Kumar D, Misra M, Dua R, Rao KVS (2010). Integration of a Phosphatase Cascade with the Mitogen-activated Protein Kinase Pathway Provides for a Novel Signal Processing Function. J Biol Chem.

[7] Barua D, Hlavacek WS, Lipniacki T (2012). A Computational Model for Early Events in B Cell Antigen Receptor Signaling: Analysis of the Roles of Lyn and Fyn. J Immunol.

[8] Healy JI, Dolmetsch RE, Timmerman LA, Cyster JG, Thomas ML, Crabtree GR, Lewis RS, Goodnow CC (1997). Different Nuclear Signals Are Activated by the B Cell Receptor during Positive Versus Negative Signaling. Immunity.

[9] Skaggs BJ, Clark MR (2004). Proximal B cell receptor signaling pathways. Signal Transduct.

[10] Kurosaki T, Hikida M (2009). Tyrosine kinases and their substrates in B lymphocytes. Immunol Rev.

[11] Geahlen RL (2009). Syk and pTyr’d: Signaling through the B cell antigen receptor. Biochim Biophys Acta.

[12] Ma H, Yankee TM, Hu J, Asai DJ, Harrison ML, Geahlen RL (2001). Visualization of Syk-antigen receptor interactions using green fluorescent protein: Differential roles for Syk and Lyn in the regulation of receptor capping and internalization. J Immunol.

[13] Veillette A, Latour S, Davidson D (2002). Negative regulation of immunoreceptor signaling. Ann Rev Immunol.

[14] Reth M, Brummer T (2004). Feedback regulation of lymphocyte signalling. Nat Rev Immunol.

[15] Andrews SF, Wilson PC (2010). The anergic B cell. Blood.

[16] Perley JP, Mikolajczak J, Buzzard GT, Harrison ML, Rundell AE (2014). Resolving Early Signaling Events in T-Cell Activation Leading to IL-2 and FOXP3 Transcription. Processes.

[17] Saltelli A, Chan K, Scott EM (2000). Sensitivity Analysis.

[18] Buzzard G (2012). Global sensitivity analysis using sparse grid interpolation and polynomial chaos. Reliab Eng Syst Saf.

