# Supplementary material for: A Computational Study of the Effects of Syk Activity on B Cell Receptor Signaling Dynamics
Source: Processes (Basel). Author manuscript; Available in PMC 2015 Oct 30. (PMC4627698; doi:10.3390/pr3010075)
Supplement: Figure S1 [file NIHMS668500-supplement-Figure_S1.pdf]

Supplementary Information

1. Sensitivity Analysis

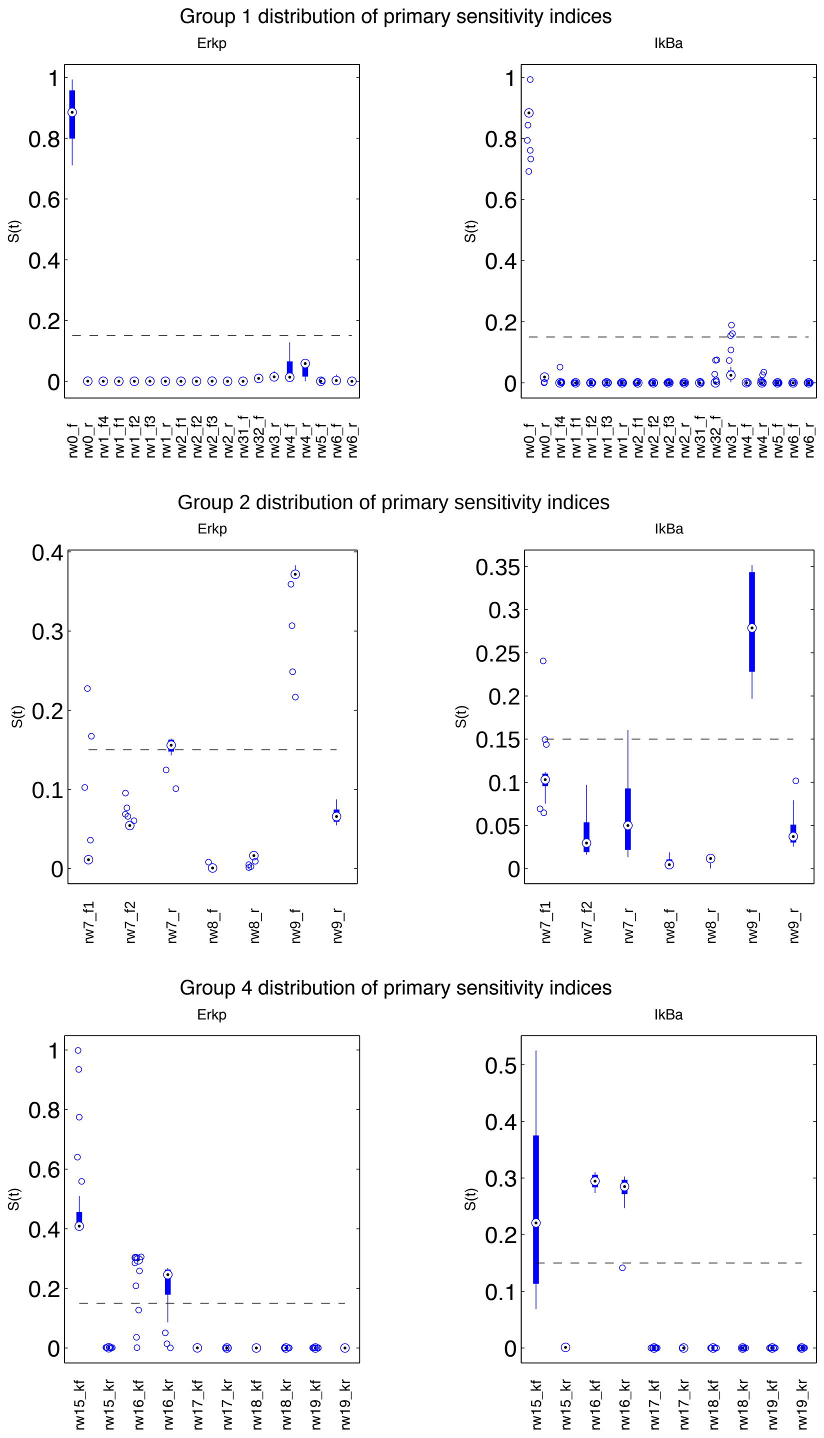

Figure S1. Cont.

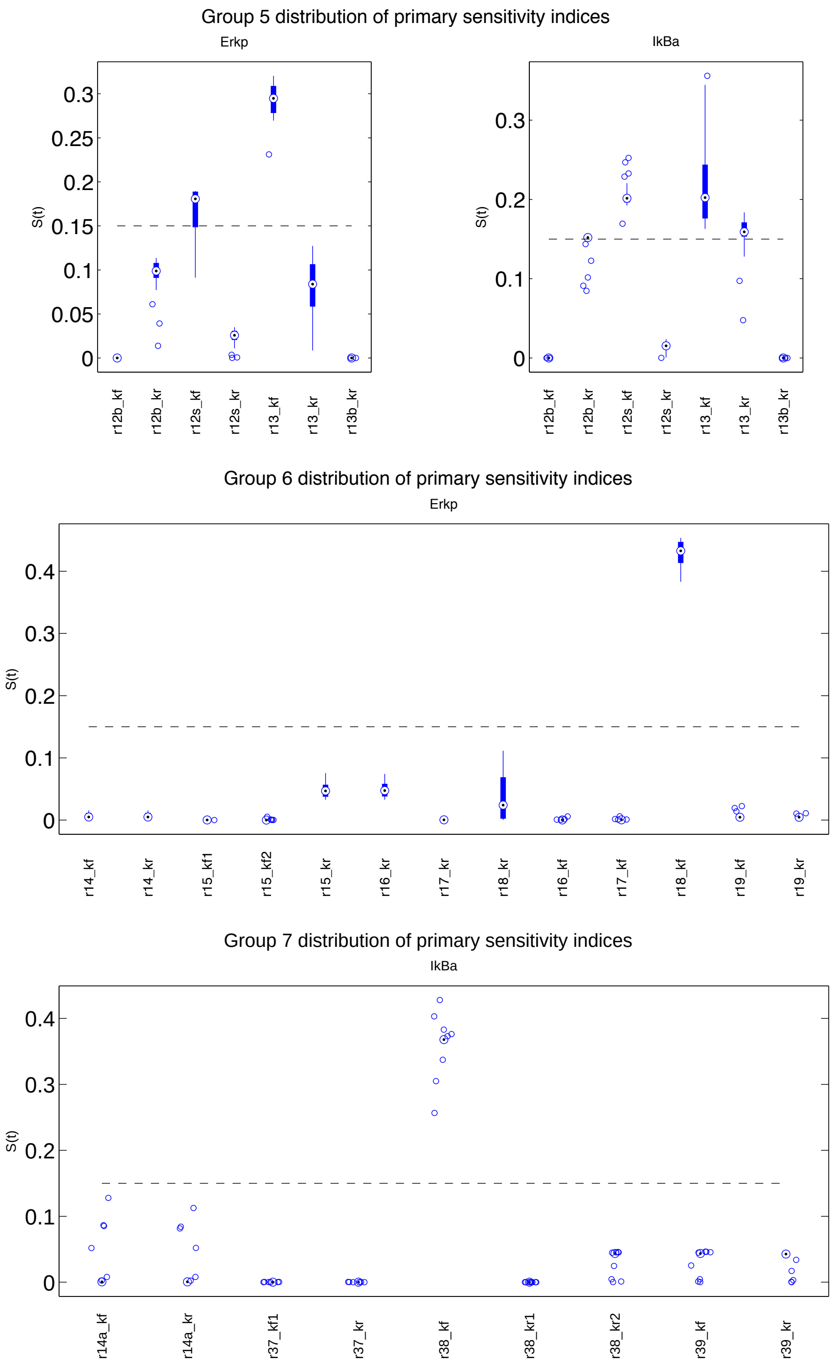

Figure S1. The results of Sobol sensitivity analysis conducted for six parameter groups. The distribution of primary Sobol indices are presented as box and whisker plots. The criterion for inclusion in the parameter screen was a median (·) sensitivity greater than 0.15. Note that there is no feedback between the Erk and NF-κB pathways and thus for group six we show the sensitivity of Erk only and for group seven we show the sensitivity of IκB only. Due to issues with stiffness, we did not conduct sensitivity analysis with respect to parameters from group three.

© 2015 by the authors; licensee MDPI, Basel, Switzerland. This article is an open access article distributed under the terms and conditions of the Creative Commons Attribution license (http://creativecommons.org/licenses/by/4.0/).
